# Treating Fatigue in Sarcoidosis—A Phenotype-Based, Treatable-Traits Approach

**DOI:** 10.3390/jcm15218346

**Published:** 2026-10-27

**Authors:** Karol Bączek, Adam J. Białas, Wojciech J. Piotrowski

**Affiliations:** Department of Pneumology, Medical University of Lodz, Kopcińskiego 22, 90-153 Lodz, Poland; karol.baczek@umed.lodz.pl (K.B.); adam.bialas@umed.lodz.pl (A.J.B.)

**Keywords:** sarcoidosis, fatigue, treatable traits, FAS, fatigue assessment scale

## Abstract

Fatigue is one of the most prevalent and debilitating symptoms in sarcoidosis, yet its mechanisms remain poorly defined and therapeutic responses are often unsatisfactory. Given its multifactorial nature and the limitations of uniform treatment strategies, there is a need for more structured and clinically actionable approaches. This narrative review summarizes current evidence on biological, behavioral, treatment-related and comorbidity-related contributors to sarcoidosis-associated fatigue and proposes an author-developed framework informed by the treatable-traits concept. Rather than defining mutually exclusive categories, the framework describes clinically recognizable fatigue patterns, including active inflammatory fatigue; treatment-related fatigue; comorbidity-related fatigue; sleep-related fatigue; neuropsychiatric, cognitive, pain and small-fiber neuropathy-related fatigue; deconditioning and lifestyle-related fatigue; and residual/post-sarcoidosis fatigue. The proposed approach emphasizes systematic identification and prioritization of one or more relevant treatable traits, followed by targeted intervention and reassessment. It is intended to support clinical reasoning rather than to serve as a validated classification. The framework integrates previously recognized contributors to fatigue into a structured clinical pathway and highlights areas in which direct evidence for fatigue-specific interventions remains limited. Prospective validation is required to determine its reproducibility, clinical utility and ability to identify patients who may benefit from trait-matched interventions.

## 1. Introduction

### 1.1. Burden of Fatigue in Sarcoidosis

Fatigue is one of the most common and disabling symptoms reported by patients with sarcoidosis. It may substantially impair daily functioning, work capacity, social participation, and health-related quality of life, even when pulmonary or extrapulmonary manifestations appear clinically stable. Therefore, fatigue should be considered a core patient-reported outcome in sarcoidosis rather than a secondary or nonspecific complaint [1,2,3].

Chronic fatigue syndrome, also known as myalgic encephalomyelitis (ME/CFS), has been described by CDC (US Centers for Disease Control and Prevention) as a combination of symptoms, such as impaired function accompanied by fatigue, post-exertional malaise, unrefreshing sleep, and cognitive impairment or orthostatic intolerance (either or both), lasting for at least 6 months [4]. So far, there is no test to confirm ME/CFS in a certain patient.

Fatigue is a frequent symptom accompanying a plethora of acute and chronic diseases, and in most cases, it does not meet the CDC definition. In the context of different chronic diseases, the term “chronic fatigue syndrome” is often used, but it is especially important to differentiate between these two terms. This situation also applies to the disease of concern, namely sarcoidosis.

Throughout this review, sarcoidosis-associated fatigue is an umbrella term. Residual fatigue denotes persistent fatigue in stable or inactive disease, while post-sarcoidosis fatigue syndrome refers more specifically to fatigue during remission. “Post-sarcoidosis chronic fatigue syndrome” is retained only in the terminology of individual publications. These expressions do not automatically imply a diagnosis of ME/CFS.

In active sarcoidosis, a variety conditions may be identified that can cause fatigue. These may include systemic inflammation, extrapulmonary sarcoidosis with special attention to neurosarcoidosis, muscle involvement, cardiac sarcoidosis and pulmonary hypertension, comorbidities (i.e., obstructive sleep apnea), the influence of anti-inflammatory and immunosuppressive drugs used to treat sarcoidosis, and psychosocial and personality features [5].

### 1.2. Why Inflammation-Centred Management Is Insufficient

Although granulomatous inflammation is an important contributor to fatigue in sarcoidosis, inflammation alone does not explain the full clinical spectrum of this symptom. Fatigue may persist despite apparent radiological or clinical remission, and in some patients, it is disproportionate to objective markers of disease activity. Conversely, persistent fatigue should not automatically be interpreted as an indication for escalation of immunosuppressive treatment [5,6,7,8].

Fatigue may persist for months or years despite apparent clinical and radiological remission of sarcoidosis. Persistent fatigue in this setting has been described as post-sarcoidosis fatigue syndrome, although its prevalence depends on the population studied and the definition applied [5,8,9]. In a study of 75 patients with sarcoidosis in clinical remission, Korenromp et al. documented substantial fatigue; 47% of the fatigued participants fulfilled the chronic fatigue syndrome criteria used in that study [9]. These findings demonstrate that clinically relevant fatigue can persist after remission, but should not be interpreted as a population-based prevalence estimate of post-sarcoidosis fatigue syndrome. The underlying mechanisms remain uncertain. Our previous cross-sectional study reported higher CRP and IL-6 concentrations in patients with persistent fatigue during sarcoidosis remission, suggesting an association with low-grade systemic inflammation without establishing causality [7].

This creates an important clinical challenge: fatigue may reflect active disease, residual immune dysregulation, treatment toxicity, sleep disorders, comorbidities, mood symptoms, pain, small-fiber neuropathy, or physical deconditioning. Therefore, an exclusively inflammation-centred approach may lead either to overtreatment with anti-inflammatory therapy or to under-recognition of other modifiable contributors [1,3,5,10,11,12].

### 1.3. Aim of This Review

The aim of this narrative review is to summarize current evidence on the assessment and management of fatigue in sarcoidosis and to propose a practical phenotype-based framework grounded in the concept of treatable traits [13,14,15]. We describe fatigue phenotypes that may support clinical reasoning, including active inflammatory fatigue, treatment-related fatigue, comorbidity-related fatigue, sleep-related fatigue, neuropsychiatric, cognitive, pain and small-fiber neuropathy-related fatigue, deconditioning and lifestyle-related fatigue, and residual/post-sarcoidosis fatigue. This framework is intended to guide individualized assessment and targeted intervention rather than to serve as a validated classification.

## 2. Methods

This article was designed as a narrative review rather than a systematic review. Relevant literature was identified through searches of PubMed/MEDLINE, Scopus and Web of Science. Search terms included combinations of “sarcoidosis”, “fatigue”, “fatigue assessment”, “treatment”, “rehabilitation”, “exercise”, “sleep”, “obstructive sleep apnoea”, “small-fiber neuropathy”, “depression”, “anxiety”, “cognition”, “glucocorticoids”, “methotrexate”, “anti-TNF”, “modafinil”, “armodafinil”, and “methylphenidate”. Additional publications were identified through reference-list screening and targeted searches for individual mechanisms and interventions.

The initial literature searches were conducted between December 2025 and March 2026. Targeted searches and reference checks for this revision were completed on 14 October 2026. The review was restricted to English-language publications. The number of records screened was not formally recorded. Study selection was undertaken by all authors (KB, AJB and WJP). Data summarised in Table 1 were extracted from full-text articles and published abstracts.

Priority was given to clinical practice guidelines, systematic reviews and meta-analyses, randomized or controlled intervention studies, and studies in which fatigue was directly assessed in patients with sarcoidosis. Observational studies were included when higher-level evidence was unavailable or when they provided information relevant to specific fatigue mechanisms or treatable traits. Evidence from other clinical populations or general guidelines was used only when direct sarcoidosis-specific evidence was limited and is identified as extrapolated evidence where relevant. No formal meta-analysis, risk-of-bias assessment, or GRADE evaluation was performed.

## 3. Assessment of Fatigue and Related Traits

### 3.1. Fatigue Questionnaires and Sarcoidosis-Specific PROs

There is no objective diagnostic test that identifies the underlying cause of sarcoidosis-associated fatigue; therefore, fatigue severity is primarily assessed using patient-reported outcome measures. Simple questioning about this symptom should be an element of medical history taking; however, it remains highly subjective. Standardized questionnaires are commonly used both in clinical practice at reference centers and in clinical research trials. The most commonly applied instrument is the Fatigue Assessment Scale (FAS) [16]. It is a patient-reported measure of fatigue severity, with primary validation data in sarcoidosis [17]. It consists of ten simple questions referring to both somatic and mental aspects of health. A total score of 22 points or more indicates fatigue, while a score of 35 or more indicates extreme fatigue [16]. The FAS is available in multiple languages. Its Croatian translation has demonstrated good reliability and validity in patients with sarcoidosis [18]. Another tool is the Multidimensional Fatigue Inventory (MFI), a 20-item self-report instrument that covers five dimensions: general fatigue, physical fatigue, mental fatigue, reduced motivation, and reduced activity [19]. This questionnaire has been used to assess fatigue in patients with cancer [20], chronically critically ill patients [21], and patients with sarcoidosis [22]. The Functional Assessment of Chronic Illness Therapy–Fatigue (FACIT-F) is a 13-item self-administered questionnaire that uses a 5-point Likert scale to assess fatigue over the previous 7 days. It has been used in many different chronic diseases [23], including sarcoidosis [24,25]. In addition, many multidimensional quality-of-life (QoL) questionnaires include the assessment of fatigue. Short Form-36 (SF-36) is a 36-question health-related QoL questionnaire, which covers three dimensions (physical, mental, and social health) and eight subscales: physical functioning, role limitations by physical problems, role limitations by emotional problems, pain, mental health, vitality, social functioning, and general health perception [26]. Sarcoidosis Health-related Quality of Life Questionnaire (SHQ) is a tool specific to the disease, which was generated from interviews with patients [27]. It covers three dimensions: daily, physical, and emotional functioning. Among 29 questions, some are related directly to fatigue [1]. King’s Sarcoidosis Questionnaire (KSQ) is a recently developed modular, multi-organ health status measure [2,28]. It consists of five modules: General health status, Lung, Medication, Skin, and Eye.

The Sarcoidosis Assessment Tool (SAT) is another validated sarcoidosis-specific patient-reported instrument and includes a fatigue module. This module was used in the study of prednisone exposure and quality of life [29,30].

### 3.2. Clinical Context: Disease Activity, Comorbidities, Sleep, Mood and Activity

Questionnaire-based fatigue assessment should be interpreted together with clinical context, including disease activity, extrapulmonary organ involvement, comorbidities, sleep quality, mood symptoms, pain, medication exposure, and physical activity. This is particularly important because similar fatigue scores may reflect different dominant mechanisms in different patients.

Fatigue has been associated with depression and anxiety [1,10,11]. Moreover, fatigue is closely associated with impaired quality of life of sarcoidosis patients [1,2,3].

Several digital biomarkers, characterizing patients’ mobility, have been proposed. Systematic review and meta-analysis focused on digital biomarkers of fatigue in different chronic diseases showed that reduced physical activity, increased sedentary behavior, and autonomic dysfunction are associated with fatigue levels across multiple diseases, though the strength of this association varies across diseases [31]. The study of severely fatigued patients showed significantly lower oxygen consumption VO2max and maximal workload in maximal cardiopulmonary exercise tests, compared to healthy controls, but the authors conclude that these biomarkers do not present higher predictive value compared to patient-reported outcome measures [32]. Strookappe et al. showed only a mild value of 6-min walk distance expressed as a percentage of the predicted value in the prediction of fatigue, explaining only 11% of reported fatigue [33].

Due to the need to objectify the tools of fatigue assessment, Kettenbach et al. identified patients with fatigue by Multidimensional Fatigue Inventory and assessed their working memory and sustained attention with functional magnetic resonance imaging. It appeared that inadequate adjustment of brain activation with increasing task difficulty is associated with chronic fatigue [22].

### 3.3. Minimal Clinically Important Difference (MCID) and Reassessment

Reassessment is essential because fatigue may change over time in response to disease activity, treatment exposure, rehabilitation, sleep interventions, or management of comorbidities. In sarcoidosis, the Fatigue Assessment Scale can be used longitudinally, and a minimal clinically important difference of approximately 4 points has been proposed to help distinguish clinically meaningful change from measurement variability. This makes repeated fatigue assessment useful both in routine care and in clinical studies evaluating phenotype-matched interventions [34].

## 4. Treatable Fatigue Phenotypes in Sarcoidosis

### 4.1. Active Inflammatory Fatigue

In patients with active sarcoidosis, granulomatous inflammation may contribute to fatigue. Anti-inflammatory treatment should follow the usual indications for sarcoidosis therapy, while other contributors to fatigue are assessed in parallel.

Oral glucocorticoids (GCs) remain first-line therapy for clinically significant symptomatic disease, using the lowest effective starting dose with a structured taper toward a minimal maintenance dose and, when possible, withdrawal [35,36]; randomized data suggest 40 mg is not superior to 20 mg prednisolone for relapse or treatment failure, changes in forced vital capacity (FVC), health status measured using SHQ and fatigue measured using FAS, supporting dose-minimisation strategies [37].

Because prolonged GC exposure contributes substantially to treatment burden and patient-reported impairment, second-line steroid-sparing immunosuppression is recommended when disease relapses during tapering, requires unacceptably high GC doses, or GC toxicity emerges; methotrexate has the strongest evidence base (including a double-blind randomized trial), while azathioprine is a common alternative with broadly comparable steroid-sparing efficacy in comparative observational data [38,39].

In selected refractory active disease, anti-TNF therapy, including infliximab or adalimumab, may be considered according to established sarcoidosis indications [36,40,41,42,43].

Importantly, the evidence supporting these anti-inflammatory therapies relates predominantly to control of sarcoidosis activity and organ involvement rather than to fatigue as a primary treatment outcome. Their inclusion in this phenotype therefore reflects treatment of the presumed underlying inflammatory driver and should not be interpreted as direct evidence of efficacy against sarcoidosis-associated fatigue.

### 4.2. Treatment-Related Fatigue

The treatment-related fatigue phenotype, also described as “steroid-related” or “iatrogenic” fatigue, refers to a clinical pattern in which fatigue is driven primarily by treatment effects rather than by ongoing granulomatous inflammation. In sarcoidosis, fatigue is common and multifactorial, but major reviews emphasise that fatigue may also be a direct consequence of therapy, particularly chronic systemic glucocorticoids, which can create or amplify fatigue even when organ disease appears stable [8].

Medication review should prioritise sarcoidosis therapy, particularly glucocorticoids and methotrexate, and symptom-directed treatments discussed in this review, such as medicines for sarcoidosis-associated neuropathic pain. Their possible contribution should be assessed using indication, dose, timing, interactions and symptom chronology. Treatment of neuropathic pain may reduce pain while introducing sedation or treatment-related fatigue; both outcomes should therefore be reassessed. Other prescribed and non-prescribed medicines should be reviewed individually when clinically relevant.

Potential contributors to treatment-related fatigue include glucocorticoid-induced proximal muscle weakness, sleep disturbance and metabolic adverse effects [44,45]. Their presence should be assessed alongside disease activity rather than inferred from glucocorticoid exposure alone. In particular, fatigue developing during treatment may reflect persistent sarcoidosis, treatment toxicity, or an unrelated comorbidity.

In the propensity-adjusted analysis, prednisone exposure exceeding 500 mg during the preceding year was associated specifically with poorer scores in the SAT fatigue domain (*p* < 0.0001) [29]. However, an exploratory post-hoc analysis suggested that cardiac sarcoidosis might explain the differences between exposure groups [29]. Residual confounding by indication therefore limits attribution of these findings to glucocorticoid toxicity.

Management should balance disease control against treatment burden. Glucocorticoid dose reduction or introduction of a steroid-sparing agent may be appropriate when clinically indicated, but should not be assumed to improve fatigue. In the PREDMETH trial, fatigue was among the most common adverse events reported with methotrexate, whereas weight gain, insomnia and increased appetite were among the most common adverse events with prednisone [45]. Fatigue should therefore be reassessed after treatment changes.

For patients with suspected glucocorticoid-induced myopathy, following screening for post-exertional malaise (PEM), individually adapted resistance exercise and rehabilitation may be considered to improve strength and function. A small study in prednisone-treated patients reported improvements in muscle mass and performance after physical training; this evidence is extrapolated and does not establish efficacy against sarcoidosis-associated fatigue [46]. Coexisting insomnia, suspected obstructive sleep apnoea and metabolic complications should be evaluated and managed on their own indications. Their treatment should not be presented as an established intervention for steroid-related fatigue in sarcoidosis. Fatigue emerging or worsening during glucocorticoid tapering should also prompt consideration of glucocorticoid withdrawal syndrome and adrenal insufficiency [47].

### 4.3. Sleep-Related Fatigue

The sleep-related fatigue phenotype describes a clinical pattern in which non-restorative sleep, sleep fragmentation, sleep-disordered breathing, circadian misalignment or sleep-related movement disorders may contribute to fatigue. These disorders may coexist with sarcoidosis and should not automatically be attributed to active granulomatous inflammation. In observational work, sarcoidosis patients report clinically relevant sleep disturbance that correlates with fatigue, mood symptoms, and poorer quality of life, supporting sleep as an important and potentially treatable contributor to the fatigue burden [48].

Several lines of evidence suggest that sarcoidosis can contribute to obstructive sleep apnoea (OSA) and other sleep disorders through disease-specific mechanisms, beyond the usual population risk factors. A focused review on sleep-disordered breathing in sarcoidosis proposes three main pathways: (i) increased upper-airway resistance due to sarcoid involvement of the upper respiratory tract, (ii) corticosteroid-related weight gain that increases pharyngeal collapsibility, and (iii) parenchymal lung involvement that may alter upper-airway mechanics and gas exchange, thereby facilitating sleep-disordered breathing and amplifying daytime symptoms such as fatigue [49].

Obstructive sleep apnoea (OSA) is a central mechanism in this phenotype because it produces non-restorative sleep and daytime impairment that can be indistinguishable from “sarcoidosis fatigue” without targeted evaluation. Clinically, this phenotype is suggested by loud snoring, apnoeas, nocturnal choking/gasping, morning headaches, and excessive daytime sleepiness. STOP-Bang may support estimation of OSA risk, but its validation predominantly concerns populations without sarcoidosis [50]. Low scores or a normal Epworth Sleepiness Scale (ESS) should not override persistent clinical suspicion. One referral-centre cohort reported OSA in 88.2% of participants and found ESS unreliable for screening [51], supporting consideration of sleep testing at a low clinical threshold without establishing a population-wide testing strategy. Diagnostic confirmation should follow sleep-medicine standards: polysomnography or technically adequate home sleep apnoea testing for uncomplicated adult cases with symptoms suggestive of moderate-to-severe OSA, with polysomnography recommended when a home test is negative or inconclusive despite persistent suspicion [52]. A case report described clinically significant OSA associated with granulomatous upper-airway infiltration. Treatment with systemic corticosteroids and initial CPAP was followed by symptomatic improvement and normalisation of a subsequent sleep study without CPAP [53]. Fatigue reductions were reported after continuous positive airway pressure (CPAP) in an uncontrolled sarcoidosis cohort, but its design prevents a causal efficacy conclusion [51].

Chronic insomnia is another frequent driver, often presenting as difficulty initiating or maintaining sleep with daytime fatigue and impaired functioning; first-line management is cognitive behavioural therapy for insomnia (CBT-I), which is recommended over sole reliance on sedative medication [54].

Restless legs syndrome (RLS) and periodic limb movement disorder can fragment sleep and produce daytime fatigue even when overt sleepiness is not prominent; current guidelines emphasise systematic assessment of iron status and evidence-based symptomatic treatment when clinically significant [55]. For restless legs syndrome (RLS) and periodic limb movements (PLM), polysomnography-based work in chronic sarcoidosis identified clinically relevant PLM and frequent RLS symptoms among patients with non-restorative sleep, indicating that motor sleep disorders can meaningfully contribute to sleep fragmentation and fatigue [56]. More recently, a comparative study found that sarcoidosis patients with small fiber neuropathy (SFN) had higher prevalence and severity of RLS, together with greater fatigue and pain, supporting a neuropathic pathway linking SFN and RLS burden [12].

Finally, circadian rhythm sleep–wake disorders should be considered when the main problem is mis-timed sleep (e.g., delayed sleep phase), in which case appropriately timed light exposure and melatonin, anchored to a stable wake time, are guideline-supported strategies [57]. As a pragmatic component of the proposed assessment, we suggest reviewing medication exposure and timing in patients with sleep-related fatigue, including the temporal relationship between treatment changes, nocturnal symptoms and daytime impairment.

### 4.4. Comorbidity-Related Fatigue

Comorbidity-related fatigue refers to a clinical pattern in which fatigue is driven or amplified by coexisting diseases rather than by granulomatous inflammation itself. This phenotype should be considered when fatigue is disproportionate to objective sarcoidosis activity, persists despite stable disease, or coexists with symptoms suggesting another medical condition. Comorbidity burden is clinically relevant in sarcoidosis and has been associated with worse symptoms, impaired quality of life and fatigue [8,58,59].

The diagnostic approach should first exclude cardiopulmonary contributors. Pulmonary hypertension, cardiac disease, chronic thromboembolic disease, hypoxaemia and clinically relevant airflow obstruction may all present with fatigue, reduced exercise tolerance or dyspnoea. In selected patients, evaluation should include oxygen saturation at rest and during exertion, pulmonary function tests including DLCO, ECG, echocardiography and further cardiac or vascular assessment when clinically indicated [59,60].

Endocrine, metabolic and haematological abnormalities should also be actively screened for. Thyroid dysfunction, diabetes mellitus, obesity, adrenal dysfunction, hypercalcaemia, renal impairment, anaemia, iron deficiency and vitamin B12 or folate deficiency may mimic or amplify sarcoidosis-associated fatigue. Laboratory assessment guided by clinical findings may include complete blood count, glucose or HbA1c, TSH, creatinine/eGFR, electrolytes, calcium, liver enzymes, ferritin or transferrin saturation, vitamin B12 and folate, with additional testing guided by clinical context [61,62,63].

Pain-related, musculoskeletal and autoimmune comorbidities should be considered when fatigue is accompanied by arthralgia, myalgia, proximal weakness, chronic pain or reduced mobility. These symptoms may reflect sarcoidosis involvement, treatment toxicity, SFN, or a separate rheumatological or musculoskeletal disease. Similarly, persistent fever, night sweats, weight loss, progressive lymphadenopathy or unexplained inflammatory markers should prompt reassessment for infection, malignancy or an alternative granulomatous disorder, because sarcoidosis diagnosis requires exclusion of other causes of granulomatous disease [59,60].

### 4.5. Neuropsychiatric, Cognitive, Pain and SFN-Related Fatigue

Neuropsychiatric, cognitive, pain and small-fiber neuropathy (SFN)-related fatigue represents a phenotype in which fatigue is driven less by pulmonary abnormalities or overt inflammatory activity, and more by altered sensory processing, autonomic dysfunction, pain symptoms, and cognitive impairment [8,12]. This phenotype is clinically suggested by a mismatch between fatigue severity and objective markers of sarcoidosis activity, especially when fatigue coexists with neuropathic pain, burning or tingling sensations, allodynia, orthostatic intolerance, palpitations, sweating abnormalities, gastrointestinal dysmotility, restless legs symptoms, concentration problems, memory complaints, anxiety, or depressive symptoms [64,65,66]. The relevance of this cluster is supported by recent comparative data showing that sarcoidosis patients with SFN have higher prevalence and severity of fatigue, pain, restless legs syndrome and cognitive impairment than sarcoidosis patients without SFN, with moderate-to-strong correlations between these symptom domains [12]. Pain and autonomic dysfunction should therefore be actively assessed rather than treated as nonspecific complaints, because SFN in sarcoidosis may manifest with both sensory symptoms and dysautonomia [64,65]. In clinical and research settings, symptom-based tools such as the Small Fiber Neuropathy Screening List (SFNSL) may support the identification of patients with probable SFN-related symptom burden; however, questionnaire-based screening should be interpreted together with the clinical picture and, when needed, objective neurological assessment [66]. Cognitive symptoms are also clinically important: everyday cognitive failure has been described in sarcoidosis and appears particularly prominent in neurosarcoidosis, where fatigue and SFN symptom burden were among the strongest predictors of cognitive complaints [67,68]. Psychological factors should not be interpreted as excluding biological disease mechanisms, since fatigue, anxiety and depressive symptoms frequently overlap in sarcoidosis and may mutually amplify disability [1,10,69]. Neuroimaging studies further support the concept that chronic fatigue in sarcoidosis may involve altered central processing, including inadequate adjustment of brain activation during cognitive tasks [22].

This grouping is organisational and does not imply a shared mechanism. The psychological, cognitive, pain and SFN components should be assessed and treated separately.

### 4.6. Deconditioning and Lifestyle-Related Fatigue

As discussed previously, fatigue in sarcoidosis is increasingly recognised as a heterogeneous clinical syndrome arising from multiple interacting mechanisms. Within this conceptual framework, physical deconditioning represents one of the most biologically plausible and potentially reversible contributors to chronic fatigue. Consequently, rehabilitation-based interventions may be particularly beneficial in patients whose symptom burden is predominantly driven by this mechanism.

There is evidence that skeletal muscle weakness occurs in patients with sarcoidosis experiencing fatigue and is associated with reduced exercise capacity, impaired health status, and poorer quality of life [70]. Reduced physical activity may contribute to a self-perpetuating vicious cycle of physical deconditioning, thereby further aggravating fatigue, a concept supported by observations in sarcoidosis and established physiological principles of pulmonary rehabilitation [9,71]. Consequently, deconditioning may become not merely a consequence of fatigue but an independent mechanism capable of perpetuating symptoms. From a physiological perspective, this distinction has important therapeutic implications, as interventions targeting physical deconditioning may remain beneficial even in patients with otherwise stable sarcoidosis.

Importantly, it is essential to distinguish between sarcoid muscle involvement and other causes of muscle weakness. It is well established that prolonged systemic corticosteroid therapy may induce steroid myopathy, which itself contributes to muscle weakness and exercise intolerance. In symptomatic muscular sarcoidosis, glucocorticoids and other immunosuppressive treatments have been used in observational cohorts, but treatment responses vary across clinical patterns. These data concern management of muscular involvement and do not establish an effect on fatigue itself [72]. Careful differential diagnosis is therefore crucial before attributing fatigue solely to physical deconditioning.

Assessment may also consider habitual physical activity, sedentary behaviour, occupational demands, perceived barriers to exercise and social support. The rationale for including these factors is partly extrapolated from studies of physical-activity behaviour in populations without sarcoidosis [73,74,75]. These studies do not establish that the same factors cause sarcoidosis-associated fatigue or define a distinct fatigue phenotype. Within the proposed framework, they are considered potentially modifiable contextual factors whose contribution should be assessed individually. Whether their systematic assessment improves fatigue outcomes in sarcoidosis remains to be established.

Comprehensive pulmonary rehabilitation appears to represent a biologically plausible therapeutic strategy for patients with deconditioning-related fatigue. By improving aerobic capacity, peripheral muscle function, exercise efficiency, and self-efficacy while simultaneously reducing activity avoidance, pulmonary rehabilitation has the potential to interrupt the vicious cycle linking physical inactivity, deconditioning, and fatigue.

Before exercise-based assessment or treatment, patients should be screened for PEM, characterised by disproportionate, often delayed symptom worsening after physical or cognitive activity and prolonged recovery. When present, management should emphasise energy management and pacing rather than fixed incremental exercise programmes. This precaution is extrapolated from ME/CFS guidance; sarcoidosis-specific evidence is limited [76].

### 4.7. Residual/Post-Sarcoidosis Fatigue

Residual or post-sarcoidosis fatigue refers to persistent, clinically relevant fatigue despite remission or substantial stabilisation of sarcoidosis. This phenotype should be suspected when fatigue remains disproportionate to objective disease activity and cannot be explained by active pulmonary or extrapulmonary involvement, treatment toxicity, sleep disorders, comorbidities, depression, small-fiber neuropathy, or marked deconditioning. The concept of post-sarcoidosis fatigue syndrome was introduced to describe persistent fatigue after otherwise inactive disease, highlighting that fatigue may continue even when conventional markers of sarcoidosis activity have improved or normalised [5,8].

The mechanisms remain uncertain, but proposed contributors include post-inflammatory immune dysregulation, low-grade systemic inflammation, altered stress-axis regulation, central fatigue mechanisms, and psychological or behavioural vulnerability. Korenromp et al. described post-inflammatory fatigue in sarcoidosis in relation to personality profiles, psychological symptoms and stress hormones, whereas recent biomarker data suggest that some patients with post-sarcoidosis fatigue may show elevated peripheral inflammatory markers, including CRP and IL-6 [7,10].

Assessment should focus on confirming the residual character of this phenotype rather than assuming ongoing inflammatory activity. This requires documentation of stable or inactive sarcoidosis, repeated fatigue assessment, preferably with the Fatigue Assessment Scale, and systematic exclusion of overlapping treatable traits such as medication-related fatigue, sleep disorders, mood symptoms, pain, small-fiber neuropathy, endocrine or metabolic abnormalities, and physical deconditioning.

Residual/post-sarcoidosis fatigue is a descriptive clinical category rather than a separately identifiable biological treatable trait. Reasonable exclusion of other contributors does not require exhaustive testing. After core assessment and appropriate targeted investigations, residual fatigue may be labelled provisionally in stable or inactive disease when relevant contributors have been addressed as far as reasonably feasible.

## 5. Practical Phenotype-Based Management Algorithm

**Figure 1:**
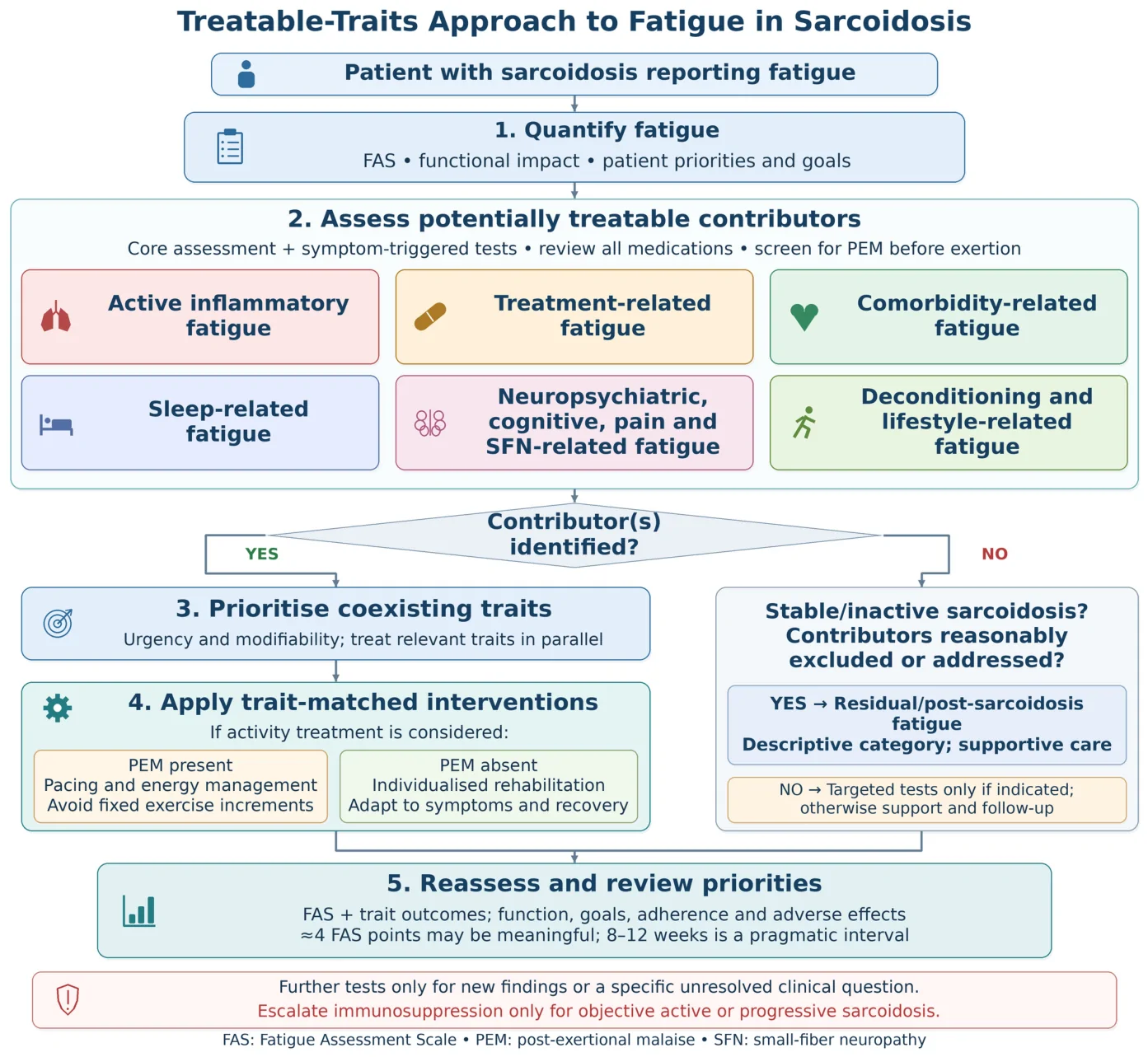
Proposed treatable-traits approach to the assessment and management of sarcoidosis-associated fatigue. Fatigue is first quantified using a validated patient-reported measure and assessed in terms of functional impact. Patients then undergo core assessment and symptom- or finding-triggered evaluation for potentially modifiable contributors, including active inflammatory fatigue, treatment-related fatigue, comorbidity-related fatigue, sleep-related fatigue, neuropsychiatric, cognitive, pain and SFN-related fatigue, and deconditioning and lifestyle-related fatigue. PEM is screened before exercise-based assessment or treatment; pacing is distinguished from progressive activity. Multiple treatable traits may coexist and should be prioritised according to clinical urgency, evidence that the trait is present, likely contribution to fatigue and modifiability. Trait-matched interventions are followed by reassessment of fatigue and trait-specific outcomes. Residual/post-sarcoidosis fatigue is a descriptive clinical category and should be considered only after stable or inactive disease has been established and other relevant treatable contributors have been reasonably excluded or addressed. Further investigation requires a specific clinical indication; otherwise symptom support and planned reassessment are appropriate. FAS: Fatigue Assessment Scale; PEM: post-exertional malaise; SFN: small-fiber neuropathy.

Fatigue in sarcoidosis should be approached as a multidimensional symptom rather than as a direct surrogate of inflammatory disease activity. A practical algorithm may help clinicians move from symptom recognition to targeted intervention by integrating disease activity, treatment burden, comorbidities, sleep disorders, neuropsychiatric symptoms, pain, small-fiber neuropathy, physical deconditioning, and residual post-inflammatory fatigue [3,5,8,12]. Treatable-Traits approach is presented in Figure 1. See Table 1 for evidence supporting phenotype-matched interventions for fatigue in sarcoidosis.

**Table 1:** Evidence supporting phenotype-matched interventions for fatigue in sarcoidosis.

Fatigue Phenotype/Treatable Trait	Main Intervention(s)	Evidence, Enrolled Population and Fatigue Outcome	Main Limitations/Interpretation
**Active inflammatory fatigue**	Glucocorticoids; methotrexate and other steroid-sparing agents; anti-TNF therapy in selected refractory disease	SARCORT [37]: open-label randomised controlled trial (RCT), *n* = 86 with pulmonary sarcoidosis; primary outcome was relapse or treatment failure, FAS secondary. Aggarwal et al. [77]: uncontrolled prospective study, *n* = 51 newly diagnosed pulmonary sarcoidosis; FAS and SHQ were main study outcomes, without a stated primary/secondary hierarchy.	Most drug studies evaluated disease control. Fatigue-specific findings are heterogeneous; an uncontrolled improvement cannot establish treatment efficacy. Inflammation control does not necessarily resolve fatigue.
**Treatment-related fatigue**	Structured review of all medications; glucocorticoid dose minimisation; management of drug-related muscle, metabolic, sleep and neuropsychiatric effects	Observational sarcoidosis studies and glucocorticoid-toxicity literature. No controlled trial selected this fatigue phenotype; a single sample size and primary fatigue outcome are not applicable to this summary.	Confounding by disease severity and cardiac involvement remains. Methotrexate and drugs used for neuropathic pain can themselves cause fatigue or sedation.
**Comorbidity-related fatigue**	Treatment of the identified comorbidity (e.g., anaemia, endocrine/metabolic disorders, cardiopulmonary disease or chronic pain)	Disease-specific guidelines and observational sarcoidosis evidence. No trial evaluated this as one fatigue-treatment package; a single sample size and primary outcome are not applicable.	The fatigue benefit depends on the contribution of the comorbidity. The proposed phenotype-matched strategy is extrapolated.
**Sleep-related fatigue (OSA)**	CPAP	Mari et al. [51]: single-group, open-label, uncontrolled cohort; *n* = 68 enrolled, 60 with OSA, 38 prescribed CPAP. Only 20 attended three-month follow-up; 13 met device-defined good adherence. FAS improvement was a within-group, exploratory outcome.	No comparator: regression to the mean, incomplete follow-up and adherence-related selection cannot be excluded. This is not controlled efficacy evidence.
**Sleep-related fatigue (insomnia, RLS or circadian disorders)**	CBT-I; correction of iron deficiency and RLS treatment; circadian interventions	Guidelines for the respective sleep disorders and observational sarcoidosis studies. No intervention trial specifically selected this fatigue phenotype; a single sample size and primary fatigue outcome are not applicable.	Treating the underlying disorder is justified, but a sarcoidosis-specific fatigue benefit remains extrapolated.
**Neuropsychiatric, cognitive, pain and SFN-related fatigue (psychological intervention)**	Online mindfulness-based cognitive therapy	TIRED [78]: open-label, multicentre RCT, *n* = 99 randomised; 93 started (46 intervention, 47 control). Stable sarcoidosis with FAS > 21; no selection for psychological symptoms. Primary outcome: between-group change in FAS at 12 weeks.	FAS difference 3.26 points (95% CI 1.18–5.33). The mean estimate is below the approximately 4-point individual-change MCID; the CI includes it. Open-label design; assignment to this phenotype is extrapolated.
**Neuropsychiatric, cognitive, pain and SFN-related fatigue (SFN and pain interventions)**	Symptomatic treatment of neuropathic pain/dysautonomia; investigational cibinetide; selected specialist therapies	Heij et al. [79]: double-blind placebo-controlled pilot in symptomatic sarcoidosis-associated SFN; 26 recruited including alternates, 22 completed (12 active, 10 placebo), multiple exploratory outcomes including FAS. Dahan et al. [80]: double-blind placebo-controlled trial, *n* = 38 with SFN; primary endpoints were neurological measures, not fatigue. Retrospective specialist-treatment data [81].	Neither trial established a fatigue-specific benefit; FAS improved similarly in both pilot groups. Neurological effects do not establish fatigue efficacy. Cibinetide remains investigational.
**Deconditioning and lifestyle-related fatigue**	Individually adapted rehabilitation and activity interventions after PEM screening; pacing if PEM is present	Meta-analysis [82]: 5 studies, *n* = 184 overall; fatigue subset: 4 studies, *n* = 146 (68 intervention, 78 control), using FAS or the Fatigue Severity Scale (FSS). Primary-outcome status varied across studies. Participants were not selected using a validated deconditioning-fatigue phenotype. Non-randomised training study [83]: *n* = 90 (49 trained, 41 untrained), fatigue among main outcomes.	Small, heterogeneous studies; uncertain pooled fatigue effect and non-randomised evidence. A rehabilitation offer as first-line supportive care is compatible with low certainty about its fatigue-specific effect. Phenotype assignment is extrapolated.
**Residual/post-sarcoidosis fatigue (neurostimulants)**	Dexmethylphenidate; armodafinil in selected specialist care	Double-blind placebo-controlled crossover trials: dexmethylphenidate [24], *n* = 10, all receiving systemic sarcoidosis treatment; armodafinil [25], *n* = 15 treated (14 completed), stable treatment and AHI < 6/h required. Fatigue measured with FAS and FACIT-F was the principal efficacy outcome in both. Neither trial selected remission-defined fatigue.	Application specifically to residual/post-sarcoidosis fatigue is extrapolated. Very small samples; cardiovascular, psychiatric and sleep-related risks require careful selection and reassessment.
**Residual/post-sarcoidosis fatigue (supportive care)**	Supportive management, pacing, sleep optimisation, psychological interventions and activity adapted to PEM	Evidence extrapolated from other fatigue contributors; no controlled trial evaluated this whole strategy in a remission-defined residual-fatigue population. A single sample size and primary fatigue outcome are not applicable.	No standardised strategy is established. Individualise support according to remaining contributors, patient goals, PEM and tolerance.

Phenotype assignments are extrapolated when the trial did not select participants for the corresponding contributor. Direct fatigue measurement does not establish a phenotype-specific mechanism or treatment effect. Sample sizes refer to enrolment unless otherwise specified; outcome hierarchy is reported as stated in the source. AHI: apnoea–hypopnoea index. Other abbreviations are defined in the abbreviation list.

### 5.1. Measure Fatigue

The first step is to actively ask about fatigue and quantify its severity using a standardized patient-reported outcome measure. The Fatigue Assessment Scale is particularly useful in sarcoidosis because it is short, validated, disease-relevant, and suitable for repeated assessment in both clinical practice and research.

### 5.2. Screen for Reversible Causes

The second step is to screen systematically for potentially reversible or treatable contributors. This should include reassessment of sarcoidosis activity, extrapulmonary organ involvement, medication burden, sleep disorders, comorbidities, mental health symptoms, pain, small-fiber neuropathy symptoms, PEM and physical deconditioning. Core assessment includes history, examination, resting oxygen saturation, review of all medications and recent sarcoidosis assessments, and basic laboratory screening including blood count, renal and liver function, calcium, glucose and TSH. Recent adequate results need not be repeated automatically. Pulmonary function testing, exertional oximetry, cardiac, neurological and muscular assessment should reflect disease monitoring needs or specific clinical findings. Iron studies, vitamin B12, folate and adrenal testing should be performed when indicated. Sleep-related symptoms should prompt screening for obstructive sleep apnoea, insomnia, restless legs syndrome, periodic limb movements and circadian rhythm disturbance.

Proposed screening criteria, confirmatory assessments, initial interventions and reassessment outcomes are summarised in Table 2. These criteria identify potentially treatable contributors but do not establish their causal contribution to fatigue.

**Table 2:** Proposed operational assessment of fatigue phenotypes and their individual contributors.

Fatigue Phenotype	Screening Instrument or Identification Criterion	Confirmatory Assessment	Initial Intervention	Trait-Specific Reassessment Outcome
Active inflammatory fatigue	Persistent, new or worsening organ-specific symptoms supported by objective disease findings. No validated fatigue-specific inflammatory threshold.	Organ-specific assessment; pulmonary function and imaging when indicated.	Guideline-directed control of active sarcoidosis.	Relevant organ function, symptoms and objective disease findings.
Treatment-related fatigue	Full medication review; symptom onset after initiation, dose change or taper. Assess sedation, proximal weakness, sleep and metabolic effects.	Clinical review of temporal relationship and alternatives; targeted muscle, metabolic or adrenal assessment.	Adjust causative medication where feasible while maintaining disease control.	Dose and adverse effects; sleep, strength or metabolic findings as relevant.
Comorbidity-related fatigue	History, examination and core laboratory results; thresholds follow the criteria for the suspected condition.	Symptom-directed tests to confirm the suspected comorbidity.	Treat the identified condition.	Relevant laboratory, physiological or functional outcome.
Sleep-related fatigue	STOP-Bang ≥ 3 suggests OSA risk [50]; low scores do not exclude OSA. ISI ≥ 10 is a screening threshold; ≥15 indicates at least moderate insomnia [84]. Clinical assessment for RLS and circadian disturbance.	Polysomnography or appropriate home sleep testing [52]; clinical confirmation of insomnia, RLS or circadian disorder.	CPAP when indicated; CBT-I for insomnia; disorder-specific management.	CPAP use and residual respiratory events; ISI or disorder-specific symptoms.
Neuropsychiatric, cognitive, pain and SFN-related fatigue	HADS-A or HADS-D ≥ 8 prompts further assessment [85]. SFNSL ≥ 11 prompts consideration of SFN; 11–48 is intermediate and >48 indicates a higher probability of abnormal temperature-threshold testing, not a diagnosis [66]. Record cognitive, pain and autonomic symptoms separately.	Clinical psychological or neurological assessment; skin biopsy, sensory or autonomic testing when indicated.	Component-specific psychological, pain or autonomic treatment; specialist assessment where needed.	HADS, SFNSL, pain interference, cognitive complaints or autonomic symptoms, as appropriate.
Deconditioning and lifestyle-related fatigue	Seven-day activity diary or wearable measure; exercise capacity or strength compared with appropriate reference values. No validated fatigue-specific activity threshold.	Consider organ or muscle disease and PEM before exertional testing.	Individually adapted rehabilitation; energy management and pacing if PEM is present.	Activity, six-minute walk distance or strength when appropriate; PEM and recovery.
Residual/post-sarcoidosis fatigue	Persistent clinically relevant fatigue (e.g., FAS ≥ 22) in stable or inactive disease after reasonable assessment and management of contributors.	Focused review after core and indicated targeted assessments; no single confirmatory test.	Supportive care, pacing, sleep and psychological support; activity adapted to PEM status.	Daily function, symptom exacerbations and patient-defined goals.

FAS should be recorded at baseline and reassessment across all phenotypes; a decrease of approximately 4 points may indicate clinically meaningful improvement [34]. Screening thresholds are not diagnostic criteria for a causal fatigue phenotype. Residual fatigue is a descriptive clinical category. Additional investigations should be guided by symptoms and findings. HADS: Hospital Anxiety and Depression Scale; HADS-A/D: anxiety/depression subscales; ISI: Insomnia Severity Index; SFNSL: Small Fiber Neuropathy Screening List. Other abbreviations are defined in the abbreviation list.

### 5.3. Identify the Dominant Phenotype

After reversible contributors have been screened, the dominant fatigue phenotype should be identified. This classification is not intended to be rigid or mutually exclusive, as many patients will have overlapping mechanisms. The aim is to identify the most clinically relevant and modifiable traits. The term “dominant phenotype” denotes a pragmatic treatment priority rather than an exclusive classification. Other identified contributors should be addressed in parallel when appropriate. When several treatable traits coexist, the dominant phenotype should be considered the factor that, based on temporal relationships, objective findings, symptom pattern, and clinical judgement, is most likely to contribute substantially to fatigue and offers a realistic therapeutic target. This prioritisation should remain provisional, as the dominant mechanism may change over time or become apparent only after treatment of competing contributors. An active inflammatory phenotype is suggested by progressive pulmonary or extrapulmonary sarcoidosis, systemic inflammatory activity, or organ-threatening disease. A treatment-related phenotype should be considered when fatigue develops or worsens during prolonged glucocorticoid exposure, particularly in the presence of proximal muscle weakness, insomnia, weight gain, metabolic complications, mood changes, or symptoms suggestive of adrenal suppression during tapering. A comorbidity-related phenotype is likely when fatigue is disproportionate to sarcoidosis activity and coexists with cardiopulmonary, endocrine, metabolic, haematological, renal, or rheumatological disease. A sleep-related phenotype should be suspected in patients with non-restorative sleep, snoring, witnessed apnoeas, morning headaches, excessive daytime sleepiness, insomnia, restless legs symptoms, or circadian misalignment. A neuropsychiatric, cognitive, pain and small-fiber neuropathy-related phenotype is suggested by neuropathic pain, dysautonomia, cognitive complaints, anxiety, depression, restless legs symptoms, or a mismatch between symptom burden and objective pulmonary impairment. A deconditioning and lifestyle-related phenotype is suggested by low physical activity, reduced exercise capacity, muscle weakness, sedentary behaviour, obesity, low self-efficacy, or fear-avoidance behaviour. Finally, residual or post-sarcoidosis fatigue should be considered only after active disease, and other treatable traits have been reasonably excluded.

### 5.4. Treat Matched Traits

Management should be directed at the dominant treatable traits rather than at fatigue as a nonspecific symptom. In patients with active inflammatory sarcoidosis, treatment should focus on appropriate disease control using the lowest effective glucocorticoid dose and steroid-sparing therapy when indicated. In treatment-related fatigue, the priority is medication review, glucocorticoid dose reduction when clinically feasible, prevention and treatment of steroid myopathy, metabolic complications, insomnia, mood symptoms, and possible adrenal insufficiency. Comorbidity-related fatigue requires targeted treatment of the underlying condition, such as pulmonary hypertension, cardiac disease, hypoxaemia, anaemia, thyroid disease, diabetes, renal dysfunction, hypercalcaemia, obesity, or chronic pain. Sleep-related fatigue should be managed according to the specific sleep disorder, including CPAP for obstructive sleep apnoea, cognitive behavioural therapy for insomnia, treatment of restless legs syndrome or periodic limb movement disorder, and correction of circadian rhythm disturbance when present. Neuropsychiatric, cognitive, pain and small-fiber neuropathy-related fatigue may require multidisciplinary management including neurological assessment, treatment of neuropathic pain and dysautonomia, psychological support, and management of anxiety or depressive symptoms. For deconditioning-related fatigue, pulmonary rehabilitation, progressive resistance and endurance training, inspiratory muscle training in selected patients, and behavioural strategies to reduce sedentary behaviour may be appropriate after PEM screening, with progression adapted to symptoms and recovery. When PEM is present, energy management and pacing should guide activity rather than fixed incremental training. In residual or post-sarcoidosis fatigue, management should be supportive and symptom-oriented, with emphasis on pacing, sleep optimization, psychological support, rehabilitation when tolerated, and avoidance of unnecessary escalation of immunosuppression in the absence of objective inflammatory activity.

### 5.5. Reassess and Escalate

Fatigue should be reassessed after a defined interval. As no validated reassessment interval has been established specifically for sarcoidosis-associated fatigue, an interval of approximately 8–12 weeks may be used as a pragmatic clinical approach, with earlier reassessment when treatment is changed or symptoms worsen. Reassessment should include repeated fatigue scoring, review of functional status, treatment adherence, adverse effects, sleep quality, physical activity, and patient-defined goals. If fatigue improves, the current phenotype-matched strategy should be continued and consolidated. If fatigue persists, the clinician should reconsider whether the dominant phenotype was correctly identified, whether several phenotypes coexist, or whether an overlooked comorbidity, sleep disorder, medication effect, pain syndrome, or neuropsychiatric contributor is present. Escalation of anti-inflammatory therapy should be reserved for patients with objective evidence of active or progressive sarcoidosis. In patients without such evidence, escalation should focus on rehabilitation, sleep medicine, neurology, psychology or psychiatry, pain management, endocrinology, or cardiopulmonary evaluation according to the suspected mechanism.

Further investigation should answer a specific unresolved clinical question or follow new findings. In their absence, management should focus on symptom support and planned reassessment rather than repeated expansion of the diagnostic battery.

## 6. Treatment Overview

There is no single treatment that is effective for all patients with sarcoidosis-associated fatigue. Management should be based on the dominant treatable phenotype, while recognising that several phenotypes may coexist in the same patient. Each intervention should have a clear therapeutic target, and its effect should be reassessed using symptoms, functional outcomes and repeated fatigue measurement. A reduction of approximately 4 points in the Fatigue Assessment Scale may be used as an indicator of clinically meaningful improvement [34]. Current treatment evidence ranges from randomised controlled trials to small observational studies, and this difference in evidence quality should be considered during shared decision-making [86].

### 6.1. Treatment of Active Inflammatory Fatigue

In the active inflammatory phenotype, treatment should follow the usual indications for sarcoidosis therapy, particularly when active disease causes organ dysfunction, progressive impairment or a major reduction in quality of life. Glucocorticoids, conventional steroid-sparing agents and, in selected refractory cases, anti-TNF therapy may control granulomatous inflammation. However, these treatments were studied mainly for control of organ disease rather than fatigue, and improvement in inflammation does not always result in improvement in fatigue.

Direct fatigue-specific data are available from an uncontrolled prospective study of 51 newly diagnosed patients with pulmonary sarcoidosis treated with oral prednisolone for six months. FAS scores and all SHQ domains improved significantly. Among the 33 patients with fatigue at baseline, 21 (63.6%) had a reduction in FAS score of more than 4 points, whereas four had a deterioration of more than 4 points. Four of the 18 patients without fatigue at baseline developed fatigue during treatment [77]. These findings suggest that fatigue may improve during treatment of pulmonary sarcoidosis, although responses are heterogeneous. The absence of an untreated comparator prevents separation of treatment effects from spontaneous improvement, and incident fatigue cannot be attributed to prednisolone alone.

In an observational study with a prospective 6-month follow-up, Elfferich et al. assessed fatigue and everyday cognitive failure in patients with sarcoidosis. The proportion receiving treatment did not differ between patients with high and normal cognitive-failure scores at baseline. Patients recently started on anti-TNF therapy showed improvement in cognitive-failure scores accompanied by a reduction in fatigue, whereas cognitive-failure scores did not improve significantly in the untreated group or in patients receiving prednisone with or without methotrexate [67]. These findings do not establish that prednisone or methotrexate worsens fatigue, and the non-randomised design limits causal interpretation of the observed improvement with anti-TNF therapy.

Persistent fatigue alone should therefore not be considered sufficient evidence of uncontrolled sarcoidosis or an indication to intensify immunosuppression [3,36].

### 6.2. Treatment of Treatment-Related Fatigue

In the treatment-related phenotype, management should begin with a structured review of all medications, while reducing treatment burden and maintaining adequate disease control. This may include using the lowest effective glucocorticoid dose, gradual withdrawal when clinically safe, or introduction of a steroid-sparing agent. Proximal muscle weakness should lead to assessment for glucocorticoid-induced myopathy and referral for strength training or rehabilitation adapted to PEM status and recovery. Steroid-related insomnia, weight gain, diabetes, mood symptoms and possible adrenal suppression should also be identified and treated. The association between higher prednisone exposure and poorer patient-reported outcomes should be interpreted cautiously because disease severity and organ involvement, particularly cardiac sarcoidosis, may confound this relationship [29]. Steroid-sparing treatment may reduce glucocorticoid toxicity, but its net effect on fatigue requires individual reassessment, as methotrexate can itself be associated with fatigue [45].

### 6.3. Treatment of Comorbidity-Related Fatigue

In the comorbidity-related phenotype, treatment should be directed at the identified condition. Relevant interventions may include correction of anaemia or iron deficiency, treatment of thyroid disease or diabetes, management of hypercalcaemia, renal or hepatic dysfunction, weight reduction in obesity, pain management and treatment of cardiopulmonary complications such as hypoxaemia, cardiac disease or pulmonary hypertension. These measures are clinically important, but they have not been studied as one standardised treatment package for fatigue in sarcoidosis. Therefore, the expected benefit depends on whether the identified comorbidity is truly contributing to the patient’s symptoms [8,86].

### 6.4. Treatment of Sleep-Related Fatigue

In the sleep-related phenotype, the specific sleep disorder should be treated. In patients with obstructive sleep apnoea, continuous positive airway pressure therapy is the main intervention. An uncontrolled prospective cohort reported within-group fatigue improvement after CPAP; 20 of 38 patients prescribed treatment underwent follow-up, and 13 of these met the device-defined adherence criterion [51]. The absence of a comparator, incomplete follow-up and adherence-related selection limit interpretation. Chronic insomnia should be managed primarily with cognitive behavioural therapy for insomnia, while iron deficiency and other aggravating factors should be addressed in patients with restless legs syndrome.

### 6.5. Treatment of Neuropsychiatric, Cognitive, Pain and SFN-Related Fatigue

In the neuropsychiatric, cognitive, pain and small-fiber neuropathy-related phenotype, treatment should address psychological symptoms, pain, cognitive difficulties and autonomic dysfunction rather than treating them as secondary complaints. The TIRED open-label randomised trial evaluated online mindfulness-based cognitive therapy in stable sarcoidosis with fatigue, without selection for psychological symptoms. The between-group FAS difference was 3.26 points (95% CI 1.18–5.33) [78]. The point estimate is below the approximately 4-point individual-change MCID, although the confidence interval includes this value and individual patients may achieve meaningful improvement. The design and effect size therefore warrant cautious interpretation. Depression and anxiety should additionally be treated according to standard psychiatric and psychological recommendations.

Management of sarcoidosis-associated small-fiber neuropathy may include standard medicines for neuropathic pain, treatment of orthostatic or autonomic symptoms and specialist neurological care. Small, randomised studies of ARA 290, later known as cibinetide, showed improvement in small-fiber neuropathy symptoms and changes in corneal nerve-fiber measures [79,80]. Improvements following intravenous immunoglobulin or anti-TNF treatment have also been reported, but mainly in retrospective observational data [81]. These therapies cannot yet be considered established treatments for fatigue itself, and cibinetide remains investigational.

### 6.6. Treatment of Deconditioning and Lifestyle-Related Fatigue

Strookappe et al. compared 49 patients completing supervised training with 41 who did not participate and found a greater fatigue reduction in the training group. The retrospective, non-randomised design and self-selected participation limit causal inference [83]. International experience-based recommendations also support individually adapted physical training in symptomatic sarcoidosis [87].

A 2023 systematic review and meta-analysis found that pulmonary rehabilitation improved exercise capacity and dyspnoea in patients with sarcoidosis, whereas the pooled effect on fatigue did not reach statistical significance [82]. However, this result should be interpreted cautiously. The fatigue meta-analysis included four studies, with 68 patients in the intervention groups and 78 controls. Two studies used the Fatigue Assessment Scale, while two used the Fatigue Severity Scale, limiting the comparability of their findings.

The interventions also differed substantially. They included structured exercise or multicomponent rehabilitation programmes, isolated inspiratory muscle training and an activity-tracker intervention with optional behavioural coaching [88,89]. Moreover, the main fatigue comparison in the activity-tracker study used a historical control group rather than a concurrently randomized control group. These differences in study design, disease severity, intervention content and fatigue measurement reduce the certainty of the pooled estimate.

The programmes evaluated by Wallaert et al. and Naz et al. most closely resembled structured multicomponent rehabilitation, although the intervention described by Naz et al. was primarily exercise-based [90,91]. Both studies reported relatively large reductions in fatigue, but their small sample sizes mean that this finding should be regarded as hypothesis-generating rather than definitive. Comprehensive pulmonary rehabilitation generally combines individualized exercise training with education and behavioural support following a detailed patient assessment [71].

Overall, the available evidence is insufficient to confirm or exclude a clinically meaningful effect of comprehensive pulmonary rehabilitation on sarcoidosis-associated fatigue. Uncertainty about the fatigue-specific effect does not preclude offering individualised rehabilitation as a first-line supportive option, consistent with the ERS guideline and Scott’s review [36,86], particularly for functional impairment in patients without PEM. Future adequately powered randomized trials should use fatigue as a predefined primary outcome, apply a standardized instrument such as the FAS, clearly describe all components of the rehabilitation programme and assess fatigue phenotypes before enrolment. This may help identify patients who are most likely to benefit from rehabilitation.

### 6.7. Treatment of Residual/Post-Sarcoidosis Fatigue

In the residual or post-sarcoidosis fatigue phenotype, treatment should begin only after stable or inactive disease has been confirmed and other relevant treatable contributors have been reasonably excluded or addressed. Mindfulness-based cognitive therapy, rehabilitation and physical activity support adapted to PEM status and recovery, and management of sleep or mood symptoms are reasonable first options. Small crossover trials reported fatigue improvement with dexmethylphenidate in 10 patients receiving systemic sarcoidosis therapy and armodafinil in 15 patients on stable therapy, with an apnoea–hypopnoea index (AHI) < 6/h required for participation [24,25]. Neither trial specifically selected remission-defined post-sarcoidosis fatigue; application to this phenotype is therefore extrapolated. Neurostimulants should therefore be reserved for carefully selected patients after assessment of cardiovascular, psychiatric and sleep-related risks, and their benefit should be reviewed regularly.

In patients with a mixed phenotype, several interventions may be required. Whenever possible, treatments should be introduced in a structured manner so that their individual effects and adverse effects can be evaluated. Lack of improvement should lead to reassessment of the original phenotype rather than automatic escalation of anti-inflammatory treatment.

## 7. Conclusions

Fatigue in sarcoidosis is not a single clinical entity with one underlying mechanism. It may be related to active inflammation, treatment toxicity, comorbidities, sleep disorders, neuropsychiatric symptoms, small-fiber neuropathy, physical deconditioning or persistent symptoms after remission. These phenotypes may overlap and may change during the disease. Therefore, fatigue severity should not be used as a direct marker of inflammatory activity without additional clinical evidence.

Direct evidence for interventions specifically targeting sarcoidosis-associated fatigue remains limited. An open-label randomised trial suggests a modest average benefit of online mindfulness-based cognitive therapy, while uncontrolled prospective evidence suggests improvement in fatigue following CPAP in patients with coexisting obstructive sleep apnoea. Exercise and rehabilitation remain biologically plausible and clinically relevant interventions, although their specific effect on fatigue is not yet established with sufficient certainty. Small crossover randomized trials suggest potential benefit from dexmethylphenidate and armodafinil in carefully selected patients with persistent fatigue, but the limited sample sizes preclude strong conclusions. Their application specifically to remission-defined fatigue is extrapolated. Therapies directed at small-fiber neuropathy require further study to establish their effects on fatigue itself. Anti-inflammatory treatment should be used when justified by active sarcoidosis and organ-related indications, rather than being prescribed or intensified solely because fatigue persists.

A practical approach should include routine questioning about fatigue, repeated assessment with a validated instrument such as the Fatigue Assessment Scale, identification of the dominant treatable phenotype, targeted treatment and planned reassessment. A change of approximately 4 FAS points may help determine whether the intervention has produced a clinically meaningful effect. The proposed phenotype-based model is a clinical framework rather than a validated classification. Future trials should classify participants according to the likely mechanism of fatigue, use fatigue as a predefined outcome and determine which treatments are most effective for specific patient groups.

Prospective evaluation could assess inter-rater agreement in identifying individual contributors, followed by a randomised comparison of trait-targeted management with usual care, following the general design used by McDonald et al. in severe asthma [92]. Outcomes should include FAS change, clinically meaningful responders, functioning, adverse effects and diagnostic burden.

## Data Availability

No new data were created or analyzed in this study. Data sharing is not applicable to this article.

## Abbreviations

The following abbreviations are used in this manuscript:

AHI	Apnoea–hypopnoea index
CBT-I	Cognitive behavioural therapy for insomnia
CDC	Centers for Disease Control and Prevention
CI	Confidence interval
CPAP	Continuous positive airway pressure
CRP	C-reactive protein
DLCO	Diffusing capacity of the lung for carbon monoxide
eGFR	Estimated glomerular filtration rate
ERS	European Respiratory Society
ESS	Epworth Sleepiness Scale
FACIT-F	Functional Assessment of Chronic Illness Therapy–Fatigue
FAS	Fatigue Assessment Scale
FSS	Fatigue Severity Scale
FVC	Forced vital capacity
GCs	Glucocorticoids
GRADE	Grading of Recommendations Assessment, Development and Evaluation
HADS	Hospital Anxiety and Depression Scale
HADS-A/HADS-D	HADS anxiety/depression subscales
HbA1c	Glycated haemoglobin
IL-6	Interleukin-6
ISI	Insomnia Severity Index
KSQ	King’s Sarcoidosis Questionnaire
MCID	Minimal clinically important difference
ME/CFS	Myalgic encephalomyelitis/chronic fatigue syndrome
MFI	Multidimensional Fatigue Inventory
OSA	Obstructive sleep apnoea
PEM	Post-exertional malaise
PET	Positron emission tomography
PLM	Periodic limb movements
PROs	Patient-reported outcomes
QoL	Quality of life
RCT	Randomised controlled trial
RLS	Restless legs syndrome
SAT	Sarcoidosis Assessment Tool
SF-36	Short Form-36
SFN	Small-fiber neuropathy
SFNSL	Small Fiber Neuropathy Screening List
SHQ	Sarcoidosis Health Questionnaire
TNF	Tumour necrosis factor
TSH	Thyroid-stimulating hormone
VO2max	Maximal oxygen uptake
WASOG	World Association for Sarcoidosis and Other Granulomatous Disorders
